# Post circumcision dorsal urethro-cutaneous fistula in pediatric male patient: A case report and literature review

**DOI:** 10.1016/j.ijscr.2024.110307

**Published:** 2024-09-24

**Authors:** Tafese Gudissa Merga, Mensur Mohammed Ahmed, Ruth Zeray, Raji Amsalu, Hiwote Girma

**Affiliations:** a Pediatric Surgery Unit, Department of Surgery, St. Paul's Hospital Millennium Medical College, Addis Ababa, Ethiopia; b Urology surgery unit, Department of Surgery, St. Paul's Hospital Millennium Medical College, Addis Ababa, Ethiopia

**Keywords:** Circumcision, Dorsal urethro-cutaneous fistula, Complication, Traditional circumcision

## Abstract

**Introduction and Importance:**

Circumcision is one of the most commonly performed procedures since ancient times for different indications. Following circumcision, various complications can happen including urethro-cutaneous fistula(UCF). The urethro-cutaneous fistula usually occurs ventrally, and our case is the first from Ethiopia to report dorsal UCF. To our knowledge, there are only two case reports with dorsal UCF.

**Case presentation:**

We report a case of a 13-year-old male child for whom UCF repair was done in June 2023, after presenting with a urine leak from the dorsal side of the penis at two sites which started after a no-health professional circumcised him. Up on evaluation, he was having dorsal UCF. He was admitted and operated on the fistula tract was excised completely, and the fistula was repaired. The patient is having a smooth post-operative course.

**Clinical discussion:**

Male circumcision is one of the most common procedures performed since ancient. It is indicated for different reasons. The incidence of circumcision varies with geography: Ethiopia is one of the countries with above 90 % rate of male circumcision. Various complications can happen following circumcision, UCF being one of the less commonly happening complications. When it happens, it will usually be on the ventral aspect, with only two cases reported having dorsal UCF. Dorsal UCF can be repaired as other types of fistula and has a similar outcome.

**Conclusion:**

Even though it is rare to find dorsal UCF it can happen following circumcision and can be managed as other UCF.

## Highlights

• UCF is a possible complication of circumcision, typically ventral, rarely dorsal.
• The fistula was successfully repaired surgically.
• Dorsal UCF, though rare, can follow circumcision and be managed similarly to other UCF types.

## 1 Introduction

Circumcision is one of the most common neonatal procedures performed since ancient times. It is a surgical procedure involving the removal of penile foreskin; after indicated for medical, cultural, or religious reasons [1]. While it is commonly performed by health professionals in the Western world, it is done by non-health personnel in developing countries like our patients [2]. After circumcision, there are different complications including Urethro-cutaneous fistula (UCF). Still, when it happens the fistula will be vent-rally located, with only two case reports having dorsal UCF after circumcision in English literature [3,4]—no report in Ethiopia making our case the first dorsal UCF report from Ethiopia. This work has been reported following the SCARE criteria.

## 2 Case presentation

**Image 1:**
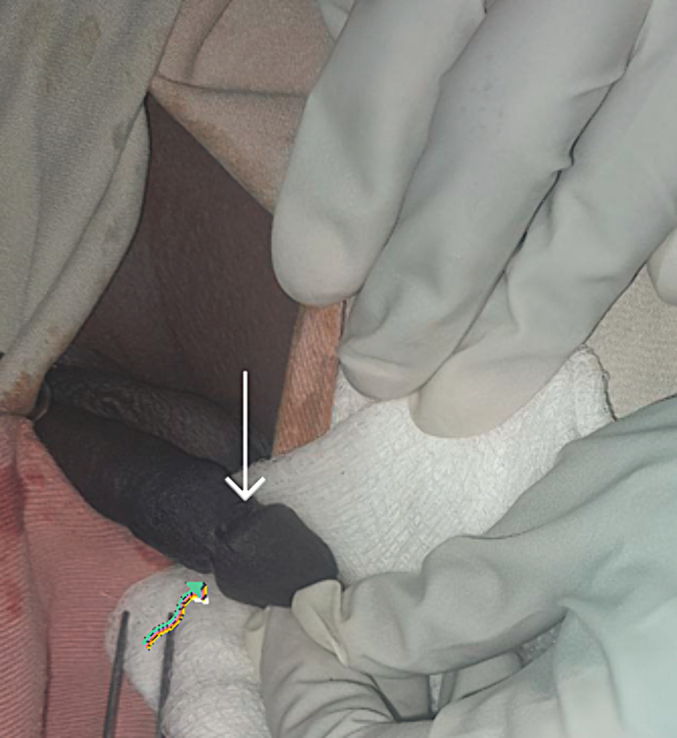
Preoperative image of the UCF sites. The white arrow points to the fistula at 2 o'clock and the fistula at 11 o'clock is pointed with a colored arrow.

**Image 2:**
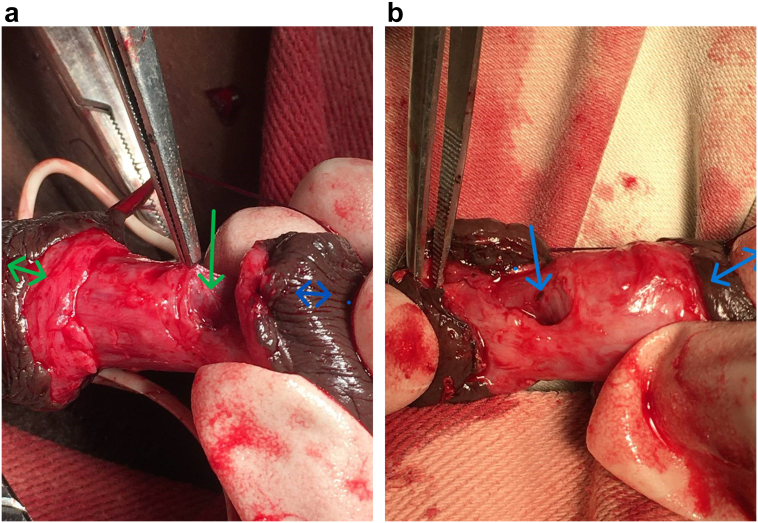
Intraoperative images of the fistula site (2A) is 11 o'clock and 2B is 2 o'clock. In image 2A, the fistula site is marked by a green arrow. The double arrows are marked toward the base (green) and glans (blue) of the penis. In image 2B the fistula site is pointed by arrow. The Site marked by anatomic pickup forceps, is the partly incised glans to see the extent of the fistula, while the double arrow marking the proximal part of the penis. (For interpretation of the references to color in this figure legend, the reader is referred to the web version of this article.)

**Image 3:**
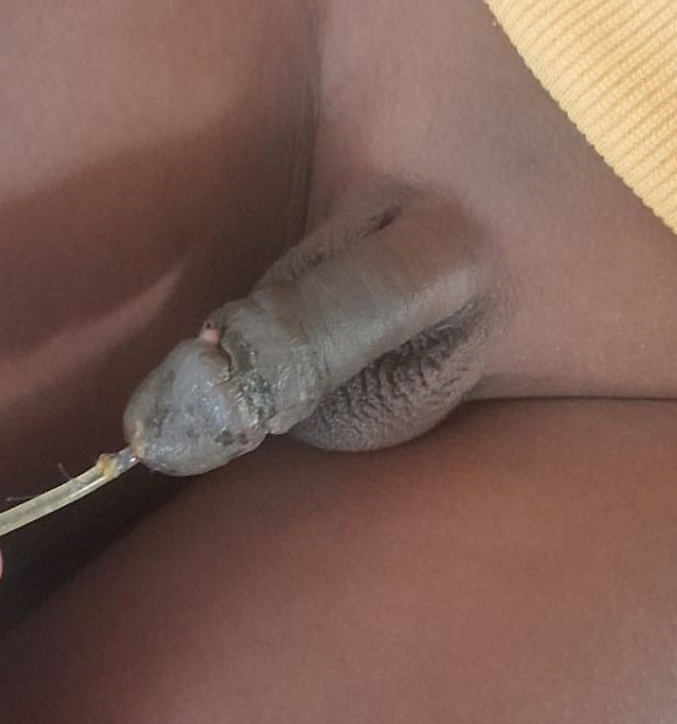
The postoperative image on the 5th post-operative day, transurethral catheter was in situ and removed after one week of the surgery.

A 13-year-old male child presented with a urine leak from the dorsal side of the penis at two sites which started after he was circumcised by a no-health professional at the age of 7 days. The family does not remember the exact time they noticed it. But it was within the first year of life. They were initially told, by the same person who did the circumcision and the community, that it would close by itself. Later they visited the local health center and got the same response. Very recently, they visited the local health center again and referred to our center. Up on evaluation, the external genitalia are well developed. There were two dorsolateral fistula sites at 2 and 11 o'clock through which the urine leaked (Image 1). The urine stream through the meatus was good, the UCF at 2 o'clock was with thin urine flow, while at 11′0cklock was dribbling. The patient was operated on. Intraoperatively, the urethra was catheterized with NG tube 10F and the fistula sites were incubated with a probe, and they were communicating the urethra from lateral. The fistula tract was excised completely (Image 2A and B), and the fistula was repaired in two layers with polyglycolic acid (PGA) 6-0. On 5th post-operative day the wound was seen healing (Image 3). The transurethral catheter was removed after a week. The child is having a smooth post-operative course at 9 months of postoperative follow-up.

## 3 Discussion

Circumcision is the surgical removal of penile prepuce. It is commonly performed for different indications like religious, cultural, and medical indications [1,5]. While the incidence is varied geographically (being commonly practiced in Muslim and Jewish communities), worldwide around 38 % of males are circumcised [6,7]. Ethiopia is one of the countries where about 90 % of males are circumcised [7,8]. The majority of circumcision is being performed by experts in the developed world, while 82 % of male circumcision in Ethiopia is performed traditionally by non-health professionals. [8,9].

There are various complications associated with circumcision including bleeding, preputio-glandular fusion, phimosis, UCF, meatal stenosis, and glandular amputation [1,[10], [11], [12]]. UCF is one of the less common circumcision complications with a wide range of occurrences of 1 % to 15 %. Different literature has revealed that the rate of complications including UCF following circumcision is higher when it is done by non-health personnel [[13], [14], [15]]. According to Ikuerowo SO, in Nigeria of the patients managed for UCF 81 % were circumcised by nurses without anesthesia, almost similar result was seen in Ghana [10,16].

UCF happens when there is communication between the urethral epithelium and skin at other openings in the urethral meatus. UCF can happen following hypospadias repair, circumcision, congenitally, or after trauma to the urethra [[17], [18], [19], [20]]. Circumcision is the second most common reason, after hypospadias repair, for the development of UCF worldwide, but according to a study done in Ibadan, Nigeria, circumcision was the most common cause of UCF accounting for 71 % [21]. In all cases, due to the anatomic location of the urethra, the UCF is located commonly vent-rally; With only two dorsal UCF cases reported worldwide in English literature until we report this case. Of these cases, one was by a non-health professional while the other was in a health center [3,4]. The management of dorsal UCF involves excision of the fistula tract and urethroplasty. The management outcome of UCF following circumcision is comparable with UCF management in other causes [22,23].

## 4 Conclusion

Even though it is rare to find dorsal UCF it can happen following circumcision and can be managed as other UCF. We recommend discouraging non-health personnel circumcision and encouraging circumcision to be done by experts.

## Consent

A written informed consent has been obtained from the patient's guardian (the mother) to have the case details and any accompanying images published. A copy of the written consent is available for review by the Editor-in-Chief of this journal on request.

## Ethical approval

Ethical approval is held to be unnecessary by St. Paul's Hospital Millennium Medical College Institutional Review Board as this is a single rare case encountered during clinical practice.

## Funding

No specific grant from funding organizations in the public, private, or nonprofit sectors was given to this manuscript.

## Author contribution

All authors contributed to different aspects. Hiwote Girma operated on the patient and also following the patient. Ruth Zeray and Raji Amsalu wrote the case presentation and wrote the draft of the case report. Tafese Gudissa and Mensur Mohammed operated on the patient and wrote the final case report and are also following the patient. All authors read and approved the final manuscript.

## Guarantor

Tafese Gudissa Merga

## Research registration number

Not applicable.

## Declaration of competing interest

No authors have disclosed any conflicts of interest.
